# Ecological Risk Assessment of Land Use Change in the Poyang Lake Eco-economic Zone, China

**DOI:** 10.3390/ijerph10010328

**Published:** 2013-01-14

**Authors:** Hualin Xie, Peng Wang, Hongsheng Huang

**Affiliations:** 1 Institute of Poyang Lake Eco-economics, Jiangxi University of Finance and Economics, Nanchang 330013, China; E-Mail: landuse2008@126.com; 2 Research Center for Agricultural Ecology of Poyang Lake Watershed, Jiangxi Agriculture University, Nanchang 330045, China; E-Mail: hhs16@vip.sina.com

**Keywords:** land use change, ecological risk assessment, land ecological management, landscape pattern, spatial statistics, Poyang Lake

## Abstract

Land use/land cover change has been attracting increasing attention in the field of global environmental change research because of its role in the social and ecological environment. To explore the ecological risk characteristics of land use change in the Poyang Lake Eco-economic Zone of China, an eco-risk index was established in this study by the combination of a landscape disturbance index with a landscape fragmentation index. Spatial distribution and gradient difference of land use eco-risk are analyzed by using the methods of spatial autocorrelation and semivariance. Results show that ecological risk in the study area has a positive correlation, and there is a decreasing trend with the increase of grain size both in 1995 and 2005. Because the area of high eco-risk value increased from 1995 to 2005, eco-environment quality declined slightly in the study area. There are distinct spatial changes in the concentrated areas with high land use eco-risk values from 1995 to 2005. The step length of spatial separation of land use eco-risk is comparatively long—58 km in 1995 and 11 km in 2005—respectively. There are still nonstructural factors affecting the quality of the regional ecological environment at some small-scales. Our research results can provide some useful information for land eco-management, eco-environmental harnessing and restoration. In the future, some measures should be put forward in the regions with high eco-risk value, which include strengthening land use management, avoiding unreasonable types of land use and reducing the degree of fragmentation and separation.

## 1. Introduction

Land use change is one of the most prominent research fields because of its key role in environment research areas including global climate change, food security, land degradation and biodiversity [1,2,3,4]. Regional land-use change leads to a variety of changes of resources and ecological processes and plays a pivotal role in regional eco-security. Land use pattern changes can cause ecological disasters because of land desertification, soil erosion, the drastic decrease of forest resources and loss of biodiversity. Ecological impacts caused by the intensification of land use have regional and cumulative characteristics and can be directly reflected in the ecosystem structure and composition. With the in-depth study of the ecological environment, ecological risk assessment has become an essential tool for the management of the ecological environment [5]. The main purpose of ecological risk assessment is to evaluate the negative impact on the likelihood and degree of harm. At this stage, the description of the ecological risk pressure has come from a single chemical factor, extended to multisource, multi-level risk factors and ecological events [6]. With the expansion of the research-scale, ecological risk assessment at the scale of the basin and the city is increasing [7,8]. Eco-risk assessment of land use describes and evaluates the likelihood and degree of harm affected by environmental pollution, human activities and natural disasters, and other sources of interference. Ecological risk assessment of land use can provide strong scientific basis for the future research on a harmonious relationship between human behavior and the ecological environment.

Humans’ exploitation activities are mainly carried out at the landscape level. Landscape is the resource and exploitation object of human activities. It also integrates human activities, ecosystem structure and function. Thus, it is chosen to be a suitable scale for research on human activities’ effects on the environment [9]. Landscape ecology emphasizes the interaction between spatial patterns, ecological processes and scale. Landscape patterns often affect ecological process (population dynamics, animal migration, biodiversity and ecological circadian condition) [10,11,12]. We can obtain a better understanding of ecological process through the study of spatial patterns, due to the interaction between patterns and processes. Therefore, eco-risk analysis based on landscape patterns can provide an integrated assessment of various types of potentially ecological impacts and cumulative consequences. Many scholars have recently studied ecological risk at the landscape level and obtained many valuable achievements [13,14,15,16].

With the wide application of the theories and methods of landscape ecology, eco-risk assessment based on landscape pattern has become one of the hotspots of regional ecosystem management. Ecological risk is a spatialized variable, and analysis of its spatial characteristics helps to reveal the mechanism and trends of ecological process. One of the obvious characteristics of landscape pattern is spatial autocorrelation [3]. A high degree of autocorrelation along a certain direction in a certain landscape pattern likely indicates some kinds of ecological process that may play a vital role [17]. The objectives of spatial statistics are to describe which patterns (e.g., random, clustered, uniform) some ecological phenomena are distributed in, and to determine if spatial proximity plays a key role in the observed distribution. At present, some methods of spatial statistic including exploratory spatial data analysis (ESDA) and semi-variance analysis are considered to be extremely effective ways for studying spatial characteristics. By way of the description and visualization of spatial information, ESDA can explore the spatial agglomeration and anomalies, and reveal the activation mechanism of the research object. Semi-variance analysis provides another way for examining autocorrelation in spatial data, which does not require second-order stationarity. Therefore, the accurate description about the spatial distribution and gradient changes of the various ecological effects can be obtained by applying ESDA and semi-variance analysis to study the eco-risk of land use.

The Poyang Lake Region of China is identified as one of the fundamental ecological function districts by the World Wide Fund for Nature (WWF). It has many ecosystem service functions, including flood water storage, climatic regulation, and degradation of pollution and others. Research on the ecological security of the Poyang Lake Region has the important ecological, economical, social and international significance. Recently, land use changes caused by the development of urbanization and industrialization have increased the landscape fragmentation in the study area. Human activities have brought enormous pressure to the ecological environment of the Poyang Lake Region. It is extremely valuable to study the ecological risk status of land use change in the Poyang Lake Eco-economic zone. In this paper, through the combination of landscape disturbance index with landscape fragmentation index, an eco-risk index is established to analyze the spatial distribution and gradient differentiation of land use eco-risk by means of the spatial autocorrelation and semi-variance methods. It can provide new thoughts and methods for sustainable land use and regionally ecological security research.

## 2. Materials and Methods

### 2.1. Study Area

**Figure 1:**
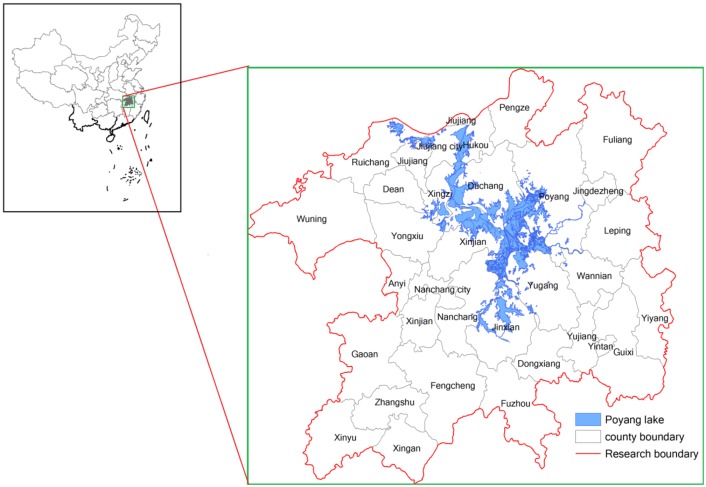
Location map of the Poyang Lake Eco-economic Zone in China.

The study area (28°30'N–30°06'N, 114°29'E–117°25'E) is located in Jiangxi Province, a southern region of China, with a surface of approximately 51,200 km^2^ (Figure 1). The area belongs to the subtropical humid climate zone, with an annual average temperature of 16~18 °C and an annual average rainfall of 1,600 mm. Annual average sunshine is about 1,473.3~2,077.5 h. Annual sunshine total radiation is about 97~114.5 Kcal/cm^2^. Soils are predominantly red soil, yellow soil and paddy soil. Poyang Lake is the largest freshwater lake in China and is one of the six wetlands with rich biodiversity in the World. Taking Poyang Lake as the core and relying on the Poyang Lake city circle, the Poyang Lake Eco-economic Zone is the significant economic zone for protecting the ecology and developing economics. The study area includes 38 counties and has a population of 20.06 million and GDP of 3,948.17 billion Yuan (RMB) in 2008. One of goals of the study area is to build an international demonstration zone for the harmonious ecological and economic development.

### 2.2. Data

Land use data of 1995 and 2005 employed in this study came from the 1:100,000 national land use database of the Data Center for Resources and Environmental Sciences, Chinese Academy of Sciences (RESDC). Landscape types in this study are divided into six classes and 22 subclasses (see Table 1). Based on the ArcGIS9.3 software, land use data resampled at the spatial resolution of 100 m × 100 m.

**Table 1:** Landscape classes of the study area.

Landscape class	Landscape subclass
Farmland landscape	Paddy field
	Dry field
Forest landscape	Wood land
	Shrub land
	Open woodland
Grass landscape	High covered pasture
	Medium covered pasture
	Low covered pasture
Water landscape	River and trench
	Lake
	Reservoir
	Lakeshore
	Lowland
Constructed landscape	City or town region
	Village residential area
	Other constructed area
Unused landscape	Sandy land
	Salted land
	Swamp
	Bare ground
	Bare rock
	Other unused area

### 2.3. Methods

Landscape spatial pattern is the outcome of the long-term action of a number of ecological and non-ecological processes. Landscape structure affects ecological processes such as the flow of energy, materials, and species between different ecosystems within a landscape. Certain strategic degradation or destruction in the landscape has an impact on the regional ecological environment [18]. In landscape ecology, indexes measuring landscape pattern mainly include diversity index, mosaic index, distance index and landscape fragmentation index [17,18,19,20]. In this paper, a disturbance index and landscape fragmentation index are constructed on the basis of common landscape indexes. Then a quantitative expression of eco-risk is established by means of the experience relationship between landscape pattern and ecological environment. Spatial characteristics of land use eco-risk in the study area are analyzed by using the method of semi-variance analysis of spatial statistics.

#### 2.3.1. Construction of Land Use Eco-Risk Index

##### Landscape Disturbance Index

Different landscape types play different roles on the maintenance of biodiversity, protection of species, improvement of the overall structure and function and the promotion of the natural structures’ succession of landscape. At the same time, different landscape types have different resistance to outside interference [21]. The landscape disturbance index (*E_i_*) is established on the foundation of landscape pattern analysis. Through the simple sum of each index, it is used to reflect the interference degree of different landscape, which is mainly determined by the human’s exploitation activities. Landscape disturbance index (*E_i_*) can be expressed by the formula below:

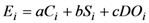
(1)
where ecological implications of the parameters can be seen in the Table 2. In Table 2, the landscape fragmentation index is used to reflect the degree of fragmentation of the divided landscape, the complexity of the structure of the landscape space, and the level of the disruption of human activities. Landscape fragmentation is considered as one of the major reasons on the loss of biodiversity and is closely related to the protection of natural resources [21]. Landscape segmentation (*S_i_*) refers to the distribution degree of the patch for a certain landscape type.

**Table 2:** Calculation method of the landscape disturbance index.

Index	Equation	Meaning of parameters	Range	Mode of acquisition
Landscape fragmentation (*C_i_*)	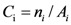	*n_i_* is the patch number of landscape *i*; *A_i_* is the total area of landscape *i.*	0~1	GIS9.3
Landscape segmentation (*S_i_*)	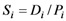	*D_i_* stands for the distance index of landscape *i*, *P_i_* is the area index of landscape *i*.	0~1	FRAGSTATS
Landscape dominance (*DO_i_*)	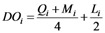	*Q_i_* is equal to the number of sampling unit with the patch *i* divided by the total number of sampling unit; *M_i_* is equal to the number of patch of landscape *i* divided by the total number of patch in the sampling unit; *L_i_* is equal to the area of landscape *i* divided by total area of sampling unit.	0~1	GIS9.3
Landscape disturbance (*E_i_*)	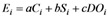	*a*, *b*, *c* represents the weight and *a* + *b* + *c* = 1.	0~1	Experts consultation method

Landscape dominance (*DO_i_*) is used to describe the extent of landscape controlled by few patch types. Its value directly reflects the degree of the patch’s effect on the formation and changes of landscape pattern. Landscape dominance is determined by the frequency, density and proportion of patch.

In Equation (1), the parameters *a*, *b*, *c* represents the weight of three indicators, respectively, and *a* + *b* + *c* = 1. The weight value reflects the degree of disturbance’s effect on ecological environment in the observed landscape. Landscape fragmentation index is considered as the most important, followed by segmentation index and dominance index. According to related research results [19,21], the weights given to the three parameters are 0.5, 0.3 and 0.2, respectively.

##### Landscape Vulnerability Index

Human activity is one of the main disturbance factors in the regional ecosystem. Land use degree not only reflects natural attributes of the land itself, but also the effect of natural factors on humans. Ecosystem in the study area is represented by six kinds of landscape types, which in a descending order of degree of vulnerability are: unused landscape, water landscape, farmland landscape, grass landscape, forest landscape, and constructed landscape. Six kinds of landscape types are respectively given to a different value: unused landscape = 6, water landscape = 5, farmland landscape = 4, grass landscape = 3, forest landscape = 2, constructed landscape = 1, then normalized to obtain their own vulnerability index (*F_i_*).

##### Land Use Eco-Risk Index

Eco-risk index of land use is constructed on the basis of the above landscape disturbance index and fragmentation index. In order to measure the relative value of integrated ecological losses in each sampling area, landscape pattern is converted into the spatialized variable of ecological risk by the sampling method. Eco-risk index (*ERI*) of land use can be expressed by the formula below:

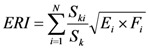
(2)
where *ERI* is the eco-risk index of land use; *N* is the number of landscape types; *E_i_* is the disturbance index of landscape *i*; *F_i_* is the vulnerability index of landscape *i*; *S_ki_* is the area of landscape components *i* in sampling unit *k*; *S_k_* is the total area of sampling unit *k*.

### 2.4. Sampling Method

**Figure 2:**
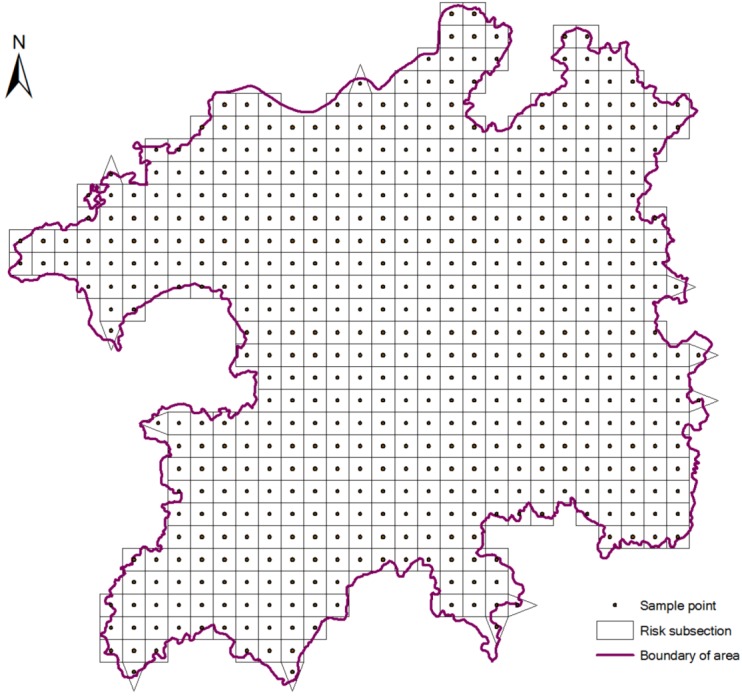
Division of the ecological risk area.

In this paper, we used equal distance sampling methods to obtain 538 units on a grid of 10 km × 10 km (Figure 2). Then we calculate the landscape pattern index for each sampling unit. According to the above established method, we obtained the result of integrated eco-risk value in each sampling unit and then take it as the eco-risk value of central point in the sampling unit.

### 2.5. Spatial Statistical Methods

#### 2.5.1. Spatial Autocorrelation Analysis

##### Global Spatial Autocorrelation

Global spatial autocorrelation can be used to measure the degree of global correlation and disparities of some social, economic and ecological phenomena. Statistic indicators measuring global spatial autocorrelation include Moran’s *I*, Geary’s *C* and Getis’s *G* [22,23,24]. Moran’s *I* is a common indicator in spatial statistics. In this article we use the Moran’s *I* to measure global spatial autocorrelation of land use ecological risk in the study area. Moran’s *I* can be expressed by the formula below:

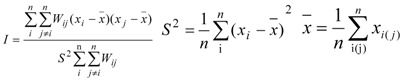
(3)
where *x_i_* is the observed value of certain attribute in the spatial unit *i*; *x_i_* is the observed value of certain attribute in the spatial unit *j*; 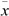 is the mean value of regional variables; *S*^2^ is the mean square deviation; *W_ij_* is the spatial weight value, which is expressed by *n* dimensional matrix *W* (n × n). The matrix is a standardized one, which can be realized by spatial distance and spatial topology.

Significance level of Moran’s *I* is commonly tested by the standardized *Z_Score_*. Its formula is as follows:

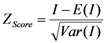
(4)


In the formula above, *E* (*I*) is the expected value of Moran’s *I*; *Var* (*I*) is the variance of Moran’s *I*. It is assumed that the *n* spatial attribute values have not spatial autocorrelation. With a significance level of 0.05, if the absolute value of *Z_Score_* is more than 1.96, the null hypothesis *H_0_* can be rejected. It shows that a significant correlation observes between the variances. The value of Moran’s *I* generally vary between 1 and −1. Under the given significance level, when Moran’s *I* value is greater than zero, positive spatial autocorrelation exists which indicates clustering state of spatially ecological phenomena. When Moran’s *I* value is less than zero, negative spatial autocorrelation exists which indicates discrete state of spatially ecological phenomena. Otherwise, spatial autocorrelation does not exist and we have a random distribution for spatially ecological phenomena.

##### Local Spatial Autocorrelation

Local indicators of spatial association (LISA) are a series of indexes decomposed directly by a global spatial autocorrelation indicator. It is expressed by the distribution state of local heterogeneity and can be used to measure the spatial disparities degree between the region *i* and its peri-regions. Local Moran’s *I* is the indicator related with global Moran’s *I* in the internal connection [22,23,24,25]. Its formula is as follows:

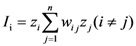
(5)
where *z_i_* is the standardization of observation value in the research unit *i*; *z_j _* is the standardization of observation value in the research unit *j*; *W*_ij_ is the spatial weight matrix. *Z_Score_* value is used to verify the significant level in the local space. Under a significant level, if *I_i_* is more than 0, spatial disparity is small between the research unit *i* and its peri-regions. Otherwise, significant disparities of land use eco-risk exist between in the research unit *i* and in its peri-regions.

Moran’s *I* scatter plot can visually reflect the spatial autocorrelation [23,25]. Under certain significant level, we can obtain the clustering map of LISA by combining the scatter plot of Moran’s *I*. The clustering map of LISA can reflect the spatial heterogeneity and can diagnose the hot spots and cold spots of land use eco-risk in the local space.

#### 2.5.2. Geo-Statistical Analysis

Based on the theory of regionalized variables with the characteristics of spatial distribution, geo-statistics is a subject to study the spatial variability and structure of natural phenomena. It can monitor, model and estimate the spatial structure of variables and is an essential part of spatial statistics [26,27,28]. Semivariance analysis is an essential component of geo-statistics. Semi-variance analysis has two main functions. Firstly, it is to be used for identifying and describing spatial structure of pattern, and the second is to be used for local optimization interpolation spatially, that is, kriging interpolation [17]. Landscape eco-risk index, as a typical regionalized variable, is provided with its spatial heterogeneity so semivariation analysis can be used to analyze it:

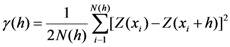
(6)
where *h* is the spatial interval distance of the two parallel sampling points, *N*(*h*) is the total numbers of sampling pairs when sampling distance is *h*, *Z*(*x_i_*) and *Z*(*x_i_ + h*) respectively stand for the observation value of eco-risk index in the spatial position *x_i_*, *x_i_ + h* (*i* = 1, 2, ..., *N* (*h*)), *N* (*h*) is the total numbers of sampling pairs when interval distance is *h*.

Semivariance is an integrated indicator for measuring spatial dependence and spatial heterogeneity. It has three key parameters: nugget, sill, range. When the interval distance *h* = 0 and *γ* (*h*) = *C_0_*, the value is named as Sill. When *h* increased to *A_0_* (Range), reached a relatively stable constant from the non-zero value, known as the Sill (*C_0_* + *C_1_*). Nugget (*C_0_*) expresses spatial heterogeneity caused by random factors and the larger value indicates that certain small-scale process cannot be overlooked. Structural variance *C_1_* denotes spatial heterogeneity caused by spatial autocorrelation part. Sill (*C_0_* + *C_1_*) indicates the greatest variation degree. The larger the Sill is, the higher spatial heterogeneity degree is. The ratio of structure variance to the Sill *C_1_*/(*C_0_* + *C_1_*) is an essential predictable measurement of variable in space [29]. While the ratio of the Nugget to Sill *C_0_*/(*C_0_* + *C_1_*) can be used to estimate the relative importance, random factors played a role on the study of spatial heterogeneity.

## 3. Results

### 3.1. Global Spatial Autocorrelation of Land Use Eco-Risk

**Figure 3:**
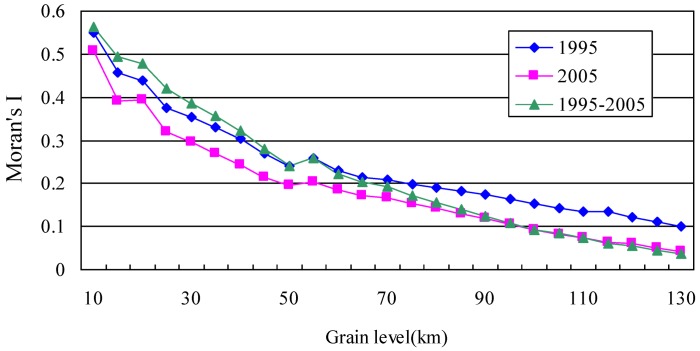
The response of Moran’s *I* for eco-risk to changes of grain size.

Moran’s *I* of eco-risk indexes at different grain level in the Poyang Lake Eco-economic Zone are listed in Table 3. From the data, we can see that Moran’s *I* of eco-risk in 1995, 2005, and from 1995 to 2000 at all grain levels are all higher than zero, which means there is a certain degree of positive correlation for eco-risk in the study area. Overall, Figure 3 also shows that Moran’s *I* of eco-risk decreased with the increase of the grain level. Spatial autocorrelation of the eco-risk from 1985 to 2000 is obvious comparable, especially the strongly positive correlation in the range of 50 km (*p* < 0.05). When the grain is smaller (<50 km), the spatial distribution of eco-risk has significant scale characteristics and dependencies. With the gradually increasing grain size, the heterogeneity of land use eco-risk sharply increases in the adjacent units and the homogeneity decreases in the research unit. On the whole, Moran’s *I* value of eco-risk in 1995 is higher than in 2000, which shows a weakly positive correlation.

**Table 3:** Moran’s I of land use eco-risk index at different grain levels in the Poyang Lake Eco-economic Zone.

Gain level (km)	1995	2005	1995–2005
10	0.5500	0.5071	0.5634
20	0.4395	0.3945	0.4792
30	0.3538	0.2952	0.3846
40	0.3029	0.2432	0.3223
50	0.2417	0.1948	0.2415
60	0.2296	0.1840	0.2228
70	0.2092	0.1662	0.1918
80	0.1895	0.1432	0.1569
90	0.1742	0.1187	0.1240
100	0.1525	0.093	0.0923
110	0.1355	0.0753	0.0731
120	0.1205	0.0595	0.0561
130	0.1006	0.0434	0.0357

### 3.2. Local Spatial Autocorrelation of Land Use Eco-Risk

**Figure 4:**
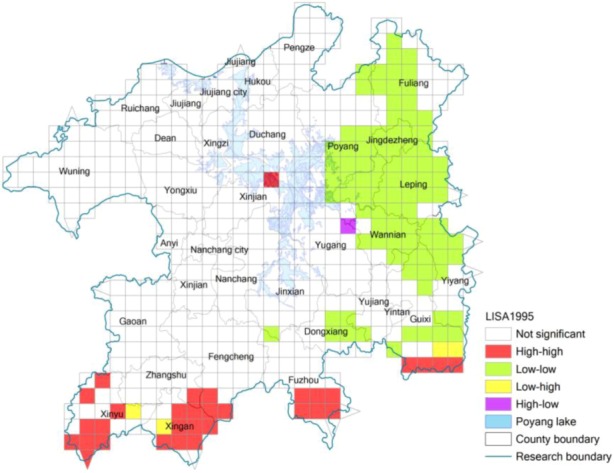
Locally spatial autocorrelation of eco-risk of the Poyang Lake Eco-economic Zone in 1995.

**Figure 5:**
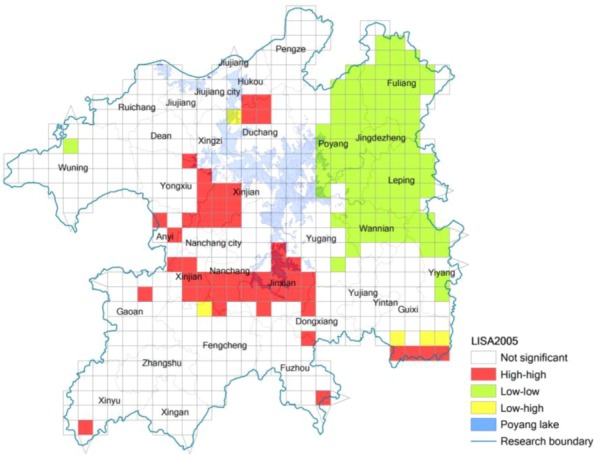
Locally spatial autocorrelation of eco-risk of the Poyang Lake Eco-economic Zone in 2005.

While a global indicator is used to measure the spatial relationship of certain phenomena in the entire study area, local indicators reflect the related extent of certain regional phenomena or geographical attribute values in a local small unit and in its adjacent units [24,30]. While the global indicator Moran’ *I* cannot validate the spatial association of eco-risk between two adjacent units, the local coefficient of spatial autocorrelation is an alternative measurement indicator [24,30]. According to Equation (3), we got the locally spatial autocorrelation value of eco-risk degree of 538 sampling units of the Poyang Lake Eco-economic Zone in 1995 and 2005 (Figure 4 and Figure 5). Comparing Figure 4 and Figure 5, we can see that there are obvious spatial changes from 1995 to 2005 about the concentrated areas with high value of land use eco-risk. Figure 4 shows that the areas with high value of land use eco-risk in 1995 were obviously concentrated in the southern region of the study area, which means there is a high value of land use eco-risk in these areas and so do in their adjacent areas.

In 2005 (see Figure 5), the areas with high value of the eco-risk were obviously concentrated in the southern and western region of Poyang Lake. All those areas are distributed around Nanchang City in 2005, where the level of urbanization and industrialization development was extremely high (*i.e.*, Xinjian County, Nanchang County and Jinxian County). This is mainly because there is rapid towns and transportation construction in those areas, which caused the higher level of landscape fragmentation and separation. The areas with low value of eco-risk in 1995 and 2005 are both obviously concentrated in the eastern Poyang Lake Region, which means that eco-risk in these areas is low, and the eco-risk in their adjacent areas is also low. Most of these areas are mountainous areas with high vegetation cover and low level of urbanization. From Figure 4 and Figure 5, there is one obvious change in the southern part of the area. This is mainly because some forest land has been developed into farmland in those areas during this period, which caused the higher level of forest landscape fragmentation and separation.

### 3.3. The Spatial and Temporal Dynamics of Land Use Eco-Risk

#### 3.3.1. Temporal Changes of Land Use Eco-Risk

**Figure 6:**
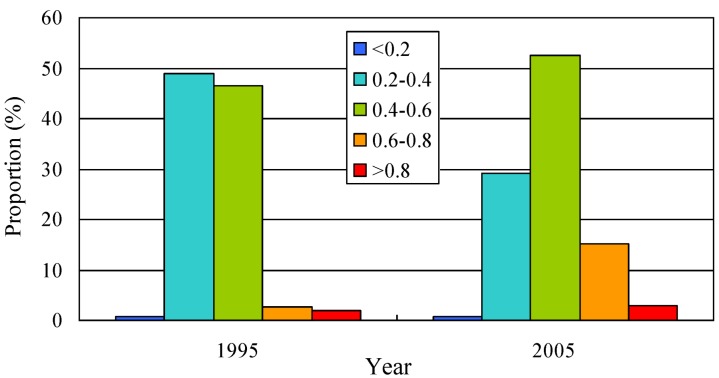
Proportion of area of land use eco-risk degree at different grade levels.

Using the method presented in Equation (2), we calculated the value of land use eco-risk in each sampling unit and carried out the analysis of classification statistics (Figure 6). Y axis in the Figure 6 represents the proportion of area of land use eco-risk degree at different grade levels. The grade (<0.2, 0.20–0.2, 0.40–0.8, >0.8) respectively represent the four levels of land use ecological risk (low, medium, relatively high and extremely high).

From the Figure 6, we can see that there is an obvious difference in the proportion of land use eco-risk at different grade levels in 1995 and 2005. In 1995, the area of land use eco-risk at the grade level (0.20–0.4) accounted for 48.88% of the total area and the proportion reduced to 21.18% in 2005, which means that there is a decreasing trend in the low eco-risk area. Although the area of land use eco-risk at the grade level (0.60–0.8) increased from 1995 to 2005, it occupied 15% of the total area in 2005. This means that land use eco-risk in the study area is still higher. In other words, the study area is in the state of ecological insecurity. The proportion of area of high levels of ecological risk (relatively high and extremely high) increased from 4.46% in 1995 to 18.22% in 2005, which means that eco-environment quality declined in the study area from the overall point of view.

#### 3.3.2. Spatial Differentiation of Eco-Risk

**Figure 7:**
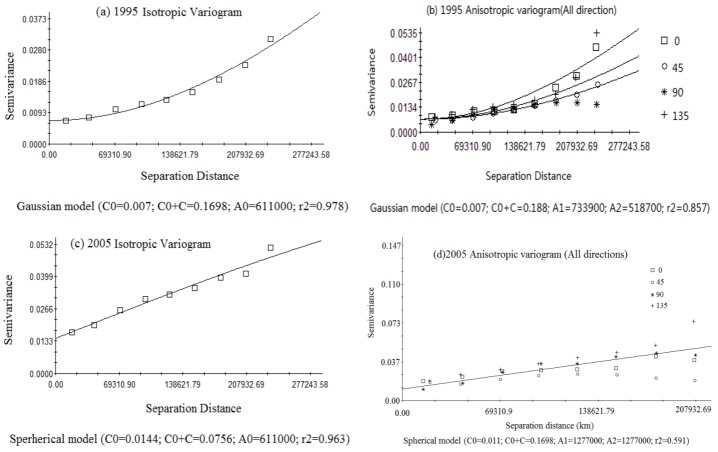
Variance function curve of eco-risk index in 1995 and 2005.

Geo-statistical methods are used to study spatial differentiation of land using eco-risk in this study. Parameters of the fitting model for land use eco-risk in 1995 and 2005 obtained by calculating the isotropic variogram of two sampling datasets (see Table 4). From Table 4, we can see that the most desirable fitting model is a Gaussian model in 1995 and a Spherical model in 2005, respectively, so the analysis of spatial structure for land use eco-risk in 1995 and 2005 were carried out by the Gaussian model and Spherical model, respectively (Figure 7). Spatial heterogeneity of land use eco-risk is mainly constituted with the random and autocorrelation part. Nugget represents the random part of spatial heterogeneity. The larger value of Nugget indicates a certain process cannot be ignored at a smaller scale. In this study, *C_0_*/(*C* + *C_0_*) in 1995 and 2005 is 4.1%, 29% respectively. This shows there are still non-structural factors at some small-scales affecting the quality of the regionally ecological environment within the selected sampling interval of 10 km, but structural factors are still leading sections for spatial differentiation in land use eco-risk.

**Table 4:** Model parameter of isotropic variogram for land use eco-risk index in 1995 and 2005.

Year	Fitting model	Nugget	Sill	Range	Effective	Proportion	r^2^	RSS
		*C_0_*	*C_0_* + *C*	Parameter *A_0_*	Range	*C*/*C_0_* + *Cssss*		
1995	Spherical model	0.00270	0.04570	611000	11000	0.941	0.898	5.190E-05
	Exponential model	0.00220	0.07650	611000	33000	0.971	0.883	5.954E-05
	Linear model	0.00277	0.02662	233949	33949	0.896	0.910	1.964E-03
	Gaussian model	0.00700	0.16980	611000	58283	0.959	0.978	1.138E-05
2005	Spherical model	0.01440	0.07560	611000	11000	0.810	0.963	3.545E-05
	Exponential model	0.01360	0.11980	611000	33000	0.886	0.961	3.866E-05
	Linear model	0.01462	0.04832	233949	33949	0.842	0.960	3.293E-05
	Gaussian model	0.02110	0.20490	555800	52673	0.897	0.930	6.686E-05

**Figure 8:**
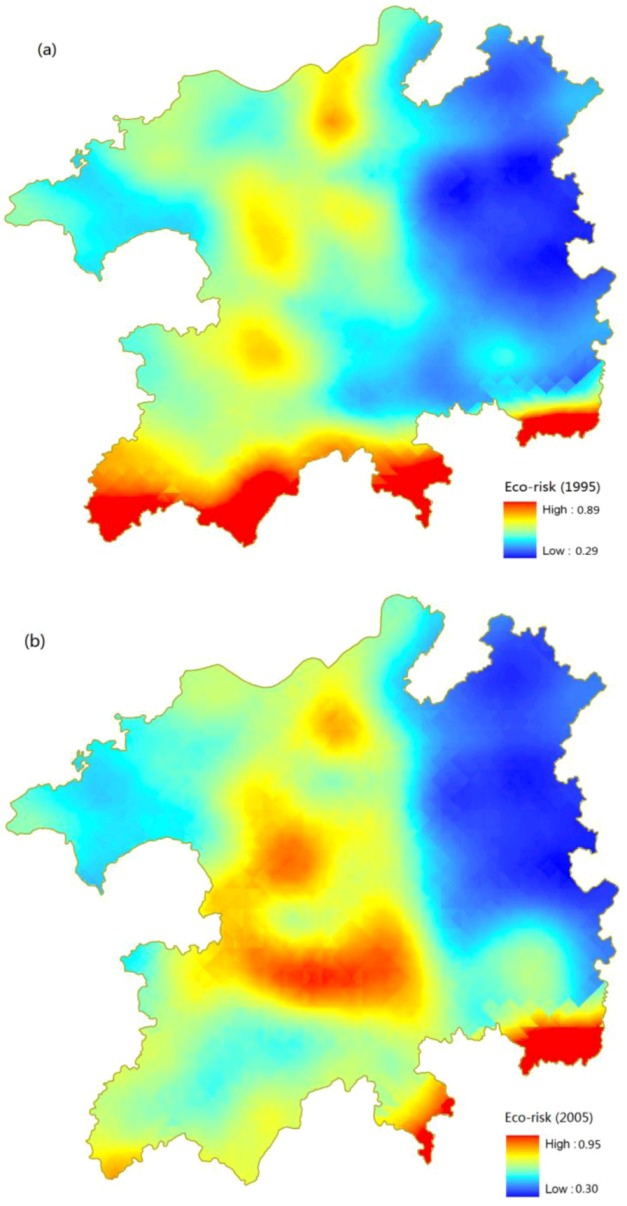
Kriging map of land use eco-risk of Poyang Lake Eco-economic Zone in 1995 and 2005.

Effective range reflects the scale of spatial autocorrelation of land use eco-risk. When the sampling distance is greater than it, various elements of land use eco-risk are random. Within this scale, spatial distribution of various elements is correlated, and its main ecological function, process, and pattern are related to the scale. From Table 4 and Figure 7, we can see that the step length of spatial differentiation of land use eco-risk is comparatively long, 58 km in 1995 and 11 km in 2005, respectively. This is mainly because differences of topographic relief are small and similarity of physiognomy is greater in the study area, which effects on the scale of spatial differentiation are not obvious. From the curves of the anisotropy variation function, we can conclude that the effects and structural factors at each direction show little difference within 100 km in 1995 and 80 km in 2005 (see Figure 7a,c). There are distinct isotropic characteristics at all directions whether in 1995 or 2005. The curve tends to deviate from the standard curve when step length is bigger than 120 km (Figure 7b) and is significant in all directions. There are some relationships between this phenomenon and the shape of the study area. Based on the theoretical model of the variogram, ecological risk pattern of land use in 1995 and 2005 were obtained by the kriging interpolated method (see Figure 8).

From Figure 8, we can see that the regions with high value of eco-risk transferred to the Northwest side of Poyang Lake from the north side of the whole study area. Low eco-risk area is largely unchanged, being mainly distributed in the eastern region. Figure 8 also shows that ecological risk is higher overall in 2005 than in 1995, which means that there is a decline in the quality of the regional eco-environment from 1995 to 2005. As can be seen from Figure 8, there is one obvious change in the southern part of the area. Contrast to in 1995, in 2005 there is no high eco-risk in the southern part of the area, which is mainly because the implementation of Returning Land from Farming to Forest Policy in that region made the poor quality farmland refund into woodland. On the other hand, it is visible from the Figure 8 that high eco-risk on the three “peninsulas” on the South-East part of the area increased, which is mainly because of the agricultural land exploitation in those regions.

## 4. Discussion and Conclusions

In this study, based on the principles of risk assessment and combined with the theory of landscape ecology and ecological risk, an assessment model of land use eco-risk is established to reflect the risk status of regional land use. 

The analysis of global spatial autocorrelation shows land use eco-risk has a strongly positive autocorrelation in the Poyang Lake Eco-economic Zone. With the increase in the grain size, the spatial autocorrelation of land use eco-risk is weakened. The analysis of local spatial autocorrelation indicates that there are spatial differences in land use eco-risk during 1995–2005. The regions with high value of the eco-risk a clearly concentrated in the top urbanized and industrialized areas. At the same time, analysis of local spatial autocorrelation also shows that land use eco-risk has obvious “cold spots”, “hot spots” and “singular point” outlier phenomenon, which quantitatively demonstrates the spatial differences and agglomeration of land use eco-risk.

Geo-statistical analysis shows that the step length of spatial differentiation of land use eco-risk is comparatively long in 1995 and 2005. There are still non-structural factors at the certain small-scale affecting on the quality of eco-environment in the Poyang Lake Eco-economic Zone. From the overall point of view, regional eco-risk was higher, and the overall eco-environment quality tended to go down during 1995–2005 in the Poyang Lake Eco-economic Zone. The research results can provide new thoughts and methods for the fields of sustainable land use and ecological security management. 

This study concludes that the policy of agricultural land exploitation makes landscape fragment and increase the ecological risk of land use. However, the implementation of Returning Land from Farming to Forest Policy increase the forest landscape connectivity and decrease the ecological risk of land use. These concludes show that land use change can affect ecological security and support the method of eco-risk assessment of land use in this study is feasible, which also can be seen by other findings [5,7,15].

In this study, the rapid development of socio-economic has made the interference of human activities on the ecological environment deepened. It can be proved by the conclusion of high value of eco-risk concentrated in the top urbanized regions. Urbanization in the study area has resulted in the loss of ecological land and the fragmentation of land pattern, which increase in the regional eco-risks. How to, reasonably, use of land resources and protect the ecological environment in the urbanized process is especially helpful and should be given sufficient attention.

With regard to research methods, ESDA and geo-statistics are effective methods measuring ecological risk pattern because they can be used to explore its distribution characteristics, local heterogeneity and homogeneity. Because eco-risk assessment based on landscape pattern can combine the horizontal interaction of land use systems and the longitudinal interaction of ecosystems, it is an effective method to study regional eco-environment. Research results can provide some help for land ecological management, ecological environmental harnessing and restoration. In the process of ecological risk prevention, some measures should be taken to control the external disturbances in the high-risk areas. For the moderate-risk areas, we should strengthen the coverage of vulnerability landscape types. The analysis of classification statistics indicates that land use eco-risk is still higher in the study area, where there are factors of ecological insecurity. In the future, regions with the high land eco-risk value in the study area should strengthen land use management, avoiding unreasonable land use types and reducing the degrees of fragmentation and separation. Only by doing this, the degree of regional eco-security of land use can be improved to promote regionally sustainable development. In the next step of our research focus, regional eco-risk analysis based on landscape pattern method needs to be improved in the evaluation index selection and the accuracy of evaluation model constructed.
